# Abdominal vs. overall obesity among women in a nutrition transition context: geographic and socio-economic patterns of abdominal-only obesity in Tunisia

**DOI:** 10.1186/s12963-015-0035-3

**Published:** 2015-01-24

**Authors:** Pierre Traissac, Rebecca Pradeilles, Jalila El Ati, Hajer Aounallah-Skhiri, Sabrina Eymard-Duvernay, Agnès Gartner, Chiraz Béji, Souha Bougatef, Yves Martin-Prével, Patrick Kolsteren, Francis Delpeuch, Habiba Ben Romdhane, Bernard Maire

**Affiliations:** 1 IRD (Institut de Recherche pour le Développement), NUTRIPASS Unit, IRD-UM-SupAgro, 911 av. Agropolis, 34394 Montpellier, France; 2 INNTA (National Institute of Nutrition and Food Technology), 11 rue Jebel Lakhdar, Bab Saadoun, 1007 Tunis, Tunisia; 3 SURVEN (Nutrition Surveillance and Epidemiology in Tunisia) Research Laboratory, 11 rue Jebel Lakhdar, Bab Saadoun, 1007 Tunis, Tunisia; 4 INSP (National Institute of Public Health), 5-7 rue de Khartoum, 1002 Tunis, Tunisia; 5 Faculty of Medicine, Tunis El Manar University, 15 rue Jebel Lakhdar, Bab Saadoun, 1007 Tunis, Tunisia; 6 ITM (Institute of Tropical Medicine), 155 Nationalestraat, 2000 Antwerp, Belgium; 7 Epidemiology and Prevention of Cardiovascular Diseases Research Laboratory, Faculty of Medicine, 15 rue Jebel Lakhdar, Bab Saadoun, 1007 Tunis, Tunisia

**Keywords:** Women, Body mass index, Waist circumference, Obesity, Abdominal obesity, Nutrition transition, Geographic disparities, Socio-economic factors, Tunisia

## Abstract

**Background:**

Most assessments of the burden of obesity in nutrition transition contexts rely on body mass index (BMI) only, even though abdominal adiposity might be specifically predictive of adverse health outcomes. In Tunisia, a typical country of the Middle East and North Africa (MENA) region, where the burden of obesity is especially high among women, we compared female abdominal vs. overall obesity and its geographic and socio-economic cofactors, both at population and within-subject levels.

**Methods:**

The cross-sectional study used a stratified, three-level, clustered sample of 35- to 70-year-old women (n = 2,964). Overall obesity was BMI = weight/height^2^ ≥ 30 kg/m^2^ and abdominal obesity waist circumference ≥ 88 cm. We quantified the burden of obesity for overall and abdominal obesity separately and their association with place of residence (urban/rural, the seven regions that compose Tunisia), plus physiological and socio-economic cofactors by logistic regression. We studied the within-subject concordance of the two obesities and estimated the prevalence of subject-level “abdominal-only” obesity (AO) and “overall-only” obesity (OO) and assessed relationships with the cofactors by multinomial logistic regression.

**Results:**

Abdominal obesity was much more prevalent (60.4% [57.7-63.0]) than overall obesity (37.0% [34.5-39.6]), due to a high proportion of AO status (25.0% [22.8-27.1]), while the proportion of OO was small (1.6% [1.1-2.2]). We found mostly similar associations between abdominal and overall obesity and all the cofactors except that the regional variability of abdominal obesity was much larger than that of overall obesity. There were no adjusted associations of AO status with urban/rural area of residence (P = 0.21), education (P = 0.97) or household welfare level (P = 0.94) and only non-menopausal women (P = 0.093), lower parity women (P = 0.061) or worker/employees (P = 0.038) were somewhat less likely to be AO. However, there was a large residual adjusted regional variability of AO status (from 16.6% to 34.1%, adjusted P < 0.0001), possibly of genetic, epigenetic, or developmental origins.

**Conclusion:**

Measures of abdominal adiposity need to be included in population-level appraisals of the burden of obesity, especially among women in the MENA region. The causes of the highly prevalent abdominal-only obesity status among women require further investigation.

## Background

Due to the epidemiological and nutrition transition, low- and middle-income countries (LMIC) have experienced a major increase in the prevalence of obesity in recent decades [1], especially in the Middle East and North Africa (MENA) where obesity, especially among women, is now a major public health challenge [2,3]. Most assessments of the burden of obesity and its variation with place of residence or socio-economic factors in LMIC rely primarily on body mass index (BMI) data [4,5]. However, in certain contexts, excess body weight may be shifting over time to greater abdominal adiposity [6]. Also, there are controversies on whether anthropometric assessments of abdominal adiposity such as waist circumference (WC) are better than BMI at predicting mortality risk [7,8]. Nevertheless, WC is now a major component of the definition of the metabolic syndrome [9] and is also among the measurements recommended for the surveillance of non-communicable diseases (NCD) by the World Health Organization [10]. However, in such nutrition transition situations, evidence pertaining to systematic comparison of abdominal vs. overall obesity and geographic or socio-economic factors based on the same large-scale data is scarce. All the more, large sample evidence regarding the variability of within-subject agreement of the two types of obesity according to place of residence or socio-economic factors is nonexistent.

Tunisia is typical of countries in the MENA region that have undergone a rapid epidemiological and nutrition transition, and today features a high prevalence of obesity, type 2 diabetes and NCDs, with close to one-third of Tunisian adults reported to be affected by the metabolic syndrome [11,12]. As observed in many countries in the region [3], the burden of obesity is especially high among women, and a third of Tunisian adult women are obese [13]. Thus, this study aimed at assessing the burden of overall and abdominal obesity, as assessed by BMI and WC respectively, and examining patterns by geographic, physiologic, and socio-economic factors. Additionally, the study quantified within-subject concordance and discordance of both types of obesity and their variations by the examined cofactors.

## Subjects and methods

### Study design and subjects

Tunisia is a relatively small country, located in North Africa between Algeria to the west and Libya to the east. It has a population of about 10 million inhabitants. It features sharp geographical contrasts, such as a long Mediterranean coastline in the north and the east vs. more mountainous and remote regions in the west, as well as marked climatic and agricultural gradients from Mediterranean in the north to desert in the south. The overall upper-middle level of development is unevenly spread across the seven administrative regions. The level of development is much higher in the northern and eastern coastal regions, including the District of Tunis around the capital city, due to industry and tourism. The western inland parts, especially the North-West and Center-West regions, which are hilly or mountainous, or the South-West region, which is mainly desert, have a much lower level of economic development.

We analyzed the subsample of women of a national cross-sectional study that surveyed Tunisian adults of both genders aged 35 to 70 from April to September 2005 [13]. The three-stage random clustered sample was stratified according to the seven administrative regions; 47 census districts were randomly selected in each region, with probability proportional to the number of households with at least one eligible subject, 20 households were then sampled in each district, and finally one 35- to 70-year-old subject was randomly selected in each household.

### Measurements and derived variables

#### Place of residence, physiological, and socio-economic factors

The urban-rural classification was that used by the Tunisian National Statistical Institute; geographic variability was studied among the seven administrative regions which compose Tunisia. Data on age, parity, menopausal status, marital status, level of education, and professional occupation of the women were collected by interview. The proxy of household welfare level was derived by multivariate analysis of items pertaining to housing characteristics and ownership of appliances: detailed analysis of the relationships between the items enabled its characterization as a continuous gradient of household “wealth”. For each household, the value of the component is a weighted average of the different items, which can be used to rank households according to increasing level of welfare either using the continuous index itself and/or as a categorical variable after recoding (in quintiles for our analyses) [14,15].

#### Overall and abdominal obesity

Standing height was measured to the mm with a stadiometer (Person-check®, Kirchner & Wilhelm, Germany), weight was measured to 100 g on a calibrated scale (Detecto, USA), WC was measured with 1-mm precision at midpoint between the lower rib and the iliac crest using a flexible tape measure [16]. We assessed overall adiposity using BMI = weight (kg)/height (m)^2^, BMI < 18.5 kg/m^2^ defined underweight, BMI ≥ 25 kg/m^2^ overweight, BMI ≥ 30 kg/m^2^ obesity (hereafter referred to as “overall obesity”) [17]. For abdominal adiposity, WC ≥ 80 cm defined increased risk abdominal adiposity, and WC ≥ 88 cm defined high-risk abdominal adiposity (hereafter referred to as “abdominal obesity”) [17].

#### Data collection

Data were collected at the subject’s home by field agents using a standardized measurement protocol and socio-demographic questionnaire.

### Data management and statistical analysis

We used Epidata 3.1 (The Epidata Association, Odense, Denmark, 2008) for data entry and validation and Stata 12 (Stata Corporation, College Station, Texas, 2011) for data management and analysis. The type I error risk was 0.05. Results are presented as estimates and standard error (in parentheses) or 0.95 confidence interval (in square brackets). For multivariate analyses, the “complete-case” analysis was used to deal with missing data. All analyses took into account the sampling design (stratification, clustering, sampling weights) [18] using *svy* Stata commands.

First, we separately quantified the burden of obesity for overall and abdominal obesity and their associations with place of residence, physiological, and socio-economic cofactors by prevalence odds-ratios (OR), estimated using logistic regression models.

Second, we studied within-subject concordance of abdominal vs. overall obesity. Beyond simply analyzing whether the two types of obesity were concordant or not (as often done when assessing agreement of two binary ratings on the same subjects), we thought it would be more informative to distinguish the two types of nonconcordance and consequently categorized as: subjects with abdominal but not overall obesity, hereafter referred to as “abdominal-only” obesity (AO); subjects with overall but not abdominal obesity, hereafter referred to as “overall-only” obesity (OO); concordant subjects (featuring both abdominal and overall obesity or neither abdominal nor overall obesity). This three-category variable was used as the response variable in multinomial regression models to compute relative prevalence ratios (RPR), to assess the relationship of AO or OO status (vs. concordance) with the place of residence as well as with physiological and socio-economic cofactors.

In both types of analyses, unadjusted associations were assessed using univariate models, while multivariate models were used to assess associations of all cofactors adjusted for one another (area, region, age, menopausal status, parity, marital status, education, profession, household welfare level).

### Ethics

The study was conducted according to the guidelines laid out in the declaration of Helsinki and all procedures involving human subjects were approved by the Ethics Committee on Human Research of the National Institute of Nutrition and the Tunisian National Council of Statistics. Informed consent was obtained from all subjects: written, or when otherwise impossible, e.g., in the case of illiteracy, their verbal consent was witnessed and formally recorded. Data were analyzed anonymously.

## Results

The response rate was 90.1% with missing data mainly due to absence or refusals, so that 2,964 women were analyzed. Two-thirds were from urban areas, their mean age was 49.1 (0.2) years, mean parity 4.7 (0.1), and half were postmenopausal. Most of the women were married, half had no formal schooling, and only a fifth had secondary education or more; three-quarters of the women had no professional activity, and less than 10% had an intermediate- or upper-level activity (Table 1). In urban vs. rural areas, parity was lower, the level of education and the level of professional activity were higher, as was household welfare level (detailed data not shown). Mean parity, the proportion of women with no schooling, and/or with no professional activity were much higher in the more rural western regions than in the more developed eastern and northern regions, while household welfare level was much lower (detailed data not shown).

**Table 1:** **35- to 70-year-old Tunisian women by place of residence, physiological, and socio-economic factors (n = 2,964)**

	n	%^a^
**Place of residence**		
**Area**	2964	
Rural	1326	33.4
Urban	1638	66.6
**Region**	2964	
South-West	406	5.3
Center-West	463	12.1
North-West	488	13.4
South-East	422	8.1
Center-East	415	21.9
North-East	397	14.1
Greater Tunis	373	25.1
**Physiological factors**		
**Age**	2964	
35-44	1033	42.4
45-54	1048	31.6
55-70	883	26.0
**Menopause**	2939	
No	1408	53.5
Yes	1531	46.5
**Parity**	2803	
1^st^ tertile (0-3)	822	37.0
2^nd^ tertile (4-5)	880	32.0
3^rd^ tertile (6+)	1101	31.0
**Socio-economic position**		
**Marital status**	2963	
Single	132	4.8
Married	2360	81.0
Divorced/widowed	471	14.2
**Education**	2963	
No formal schooling	1713	48.9
Primary school	878	31.7
Secondary or more	372	19.4
**Professional activity**	2963	
Not working/Retired	2390	76.2
Employee/worker	441	15.9
Upper/Intermediate	132	7.9
**Household welfare index** ^**b**^	2805	
1^st^ quintile	761	21.6
2^nd^ quintile	695	21.1
3^rd^ quintile	606	20.4
4^th^ quintile	415	17.7
5^th^ quintile	328	19.2

^a^Weighted proportions to account for differential probabilities of selection.

^b^Asset-based household welfare index: increasing welfare from 1^st^ to 5^th^ quintile.

Mean BMI was 28.4 (0.2) kg/m^2^. Almost three women out of four were overweight, and about 80% had WC ≥ 80 cm (Table 2). More than a third had overall obesity, and almost two-thirds had abdominal obesity. There was a significant +23.4% [21.0-25.6] difference in the national estimate of prevalence when abdominal status was used instead of overall obesity status.

**Table 2:** **Anthropometric characteristics of 35- to 70-year-old Tunisian women (n = 2,964)**

n = 2,964			Mean or %^a^	s.e.^b^	C.I.^c^
**Basic anthropometric characteristics**					
Weight (kg)			69.4	0.4	68.6-70.3
Height (cm)			156.5	0.2	156.1-156.8
Waist circumference (cm)			91.2	0.4	90.5-92.0
**Overall adiposity**					
Body mass index (kg/m^2^)			28.4	0.2	28.0-28.7
Underweight: BMI < 18.5			1.8%	0.3	1.3-2.4
Overweight: BMI ≥ 25.0			71.1%	1.3	68.5-73.6
Overall obesity: BMI ≥ 30.0			37.0%	1.3	34.5-39.6
**Abdominal adiposity**					
Increased risk: WC ≥ 80 cm			80.6%	1.0	78.6-82.6
Abdominal obesity: WC ≥ 88 cm			60.4%	1.4	57.7-63.0
**Abdominal x overall obesity**					
	**WC** ≥ **88 cm**	**BMI** ≥ **30 kg/m**^**2**^			
AO^d^	Yes	No	25.0%	1.1	22.8-27.1
OO^e^	No	Yes	1.6%	0.3	1.1-2.2
Concordant	Yes	Yes	35.4%	1.3	32.9-37.9
	No	No	38.0%	1.4	35.3-40.7

^a^Mean for interval variables, prevalence proportion for binary variables (weighted estimates accounting for unequal probabilities of selection).

^b^Standard error of estimates taking into account sampling design.

^c^P = 0.95 confidence interval taking into account sampling design.

^d^Abdominal-only obesity.

^e^Overall-only obesity.

Urban vs. rural contrasts were slightly more marked for overall than abdominal obesity, for which there was no residual association once adjusted for all other variables (Table 3). The geographic contrasts (higher prevalence in the eastern than western regions), were much more marked for abdominal than overall obesity. The association with age was similar for both types of obesity, as women over 45 were more obesity-prone. After adjustment, postmenopausal women were no more obesity-prone than premenopausal women. After adjustment, there was an increase in abdominal obesity but not in overall obesity with parity. The prevalence of both types of obesity was highest among women with a primary level of education, but once adjusted, associations with education were weak. There was a decreasing gradient of both types of obesity with a higher level of professional activity, although the gradient was less marked for overall obesity. There was a marked increase in both types of obesity with household welfare.

**Table 3:** **Overall and abdominal obesity by place of residence, physiological, and socio-economic factors among 35- to 70-year-old Tunisian women (complete-case analysis n = 2,633)**

		Overall obesity (BMI ≥ 30 kg/m^2^)					Abdominal obesity (WC ≥ 88 cm)				
		Unadjusted			Adjusted^a^		Unadjusted			Adjusted^a^	
	n	%^b^	OR^c^	C.I.^d^	OR^c^	C.I.^d^	%^b^	OR^c^	C.I.^d^	OR^c^	C.I.^d^
**Place of residence**											
**Area**			P < 0.0001		P = 0.021			P < 0.0001		P = 0.45	
Rural	1184	24.1%	1	-	1	-	49.7%	1	-	1	-
Urban	1449	45.0%	2.6	2.0-3.3	1.4	1.1-1.7	67.7%	2.1	1.6-2.7	1.1	0.9-1.5
**Region**			P < 0.0001		P < 0.0001			P < 0.0001		P < 0.0001	
South-West	366	32.9%	1	-	1	-	43.1%	1	-	1	-
Center-West	433	25.0%	0.7	0.5-1.0	1.0	0.7-1.5	42.4%	1.0	0.6-1.5	1.2	0.8-1.9
North-West	440	26.5%	0.7	0.5-1.2	1.0	0.7-1.6	42.8%	1.0	0.7-1.5	1.3	0.9-2.0
South-East	384	44.9%	1.7	1.1-2.6	1.7	1.1-2.6	77.2%	4.5	3.1-6.4	5.0	3.4-7.2
Center-East	366	39.9%	1.4	0.9-2.0	1.3	0.9-1.9	63.9%	2.3	1.6-3.4	2.5	1.7-3.8
North-East	326	35.7%	1.1	0.8-1.7	1.3	0.9-1.8	65.1%	2.5	1.7-3.6	3.1	2.1-4.7
Greater Tunis	318	49.3%	1.9	1.3-3.0	1.7	1.1-2.5	77.1%	4.4	2.7-7.4	5.4	3.5-8.2
**Physiological factors**											
**Age**			P = 0.029		P = 0.035			P < 0.0001		P = 0.018	
35-44 y.	886	34.0%	1	-	1	-	53.6%	1	-	1	-
45-54 y.	937	42.5%	1.4	1.1-1.9	1.6	1.1-2.2	66.2%	1.7	1.3-2.2	1.5	1.1-2.1
55-70 y.	810	38.6%	1.2	1.0-1.6	1.5	1.0-2.2	68.5%	1.9	1.4-2.5	1.5	1.0-2.2
**Menopause**			P = 0.96		P = 0.22			P = 0.001		P = 0.46	
No	1242	37.9%	1	-	1	-	57.5%	1	-	1	-
Yes	1391	38.0%	1.0	0.8-1.2	0.8	0.6-1.1	66.3%	1.5	1.2-1.8	1.1	0.8-1.5
**Parity**			P = 0.023		P = 0.55			P = 0.22		P = 0.019	
1^st^ tertile (0-3)	772	38.4%	1	-	1	-	58.4%	1	-	1	-
2^nd^ tertile (4-5)	819	41.6%	1.1	0.9-1.5	1.2	0.9-1.5	63.7%	1.3	1.0-1.6	1.3	1.0-1.7
3^rd^ tertile (6+)	1042	33.8%	0.8	0.6-1.1	1.0	0.7-1.4	63.4%	1.2	0.9-1.6	1.5	1.1-2.0
**Socio-economic position**											
**Marital status**			P = 0.65		P = 0.89			P = 0.63		P = 0.55	
Other	443	39.2%	1	-	1	-	65.4%	1	-	1	-
Married	2190	37.7%	0.9	0.7-1.2	1.0	0.7-1.3	61.0%	0.8	0.6-1.1	0.9	0.7-1.2
**Education**			P < 0.0001		P = 0.12			P = 0.0081		P = 0.048	
No formal schooling	1550	32.5%	1	-	1	-	59.1%	1	-	1	-
Primary school	769	45.6%	1.7	1.4-2.2	1.2	0.9-1.6	67.3%	1.4	1.1-1.8	1.1	0.9-1.5
Secondary or more	314	39.8%	1.4	1.0-1.9	0.9	0.5-1.4	59.0%	1.0	0.7-1.4	0.7	0.5-1.1
**Professional activity**			P = 0.54		P = 0.079			P = 0.035		P = 0.014	
Not working/Retired	2142	38.6%	1	-	1	-	63.9%	1	-	1	-
Employee/worker	376	38.0%	1.0	0.7-1.4	1.0	0.7-1.5	54.9%	0.7	0.5-1.0	0.7	0.5-1.0
Upper/Intermediate	115	31.9%	0.7	0.4-1.3	0.5	0.3-0.9	53.5%	0.7	0.4-1.1	0.5	0.3-0.9
**Household welfare index** ^**e**^			P < 0.0001		P < 0.0001			P < 0.0001		P < 0.001	
1^st^ quintile	710	17.0%	1	-	1	-	41.8%	1	-	1	-
2^nd^ quintile	657	33.6%	2.5	1.8-3.5	1.9	1.4-2.7	59.4%	2.0	1.5-2.7	1.6	1.2-2.1
3^rd^ quintile	568	45.3%	4.0	2.9-5.6	2.8	2.0-4.0	70.0%	3.1	2.3-4.1	2.2	1.6-3.0
4^th^ quintile	385	50.5%	5.0	3.5-7.0	3.5	2.4-5.2	72.8%	3.7	2.7-5.3	2.7	1.9-3.9
5^th^ quintile	313	46.9%	4.3	2.9-6.4	3.6	2.3-5.7	68.2%	3.0	1.9-4.7	2.8	1.9-4.3

^a^Association of response variable with each place of residence, physiological, or socio-economic variable adjusted for all other variables in column 1.

^b^Prevalence proportion (weighted estimates).

^c^Prevalence Odds-Ratio vs. reference category for which OR = 1, taking into account sampling design.

^d^0.95 confidence interval taking into account sampling design.

^e^Increasing household welfare level from 1^st^ to 5^th^ quintile.

At the subject level, abdominal and overall obesity status was concordant for 73.4% [71.1-75.6] of the women; only 1.6% [1.1-2.2] had overall-only obesity (OO), while 25.0% [22.8-27.1] of women had abdominal-only obesity (AO) (Table 2). There were no urban vs. rural differences in the proportion of AO (Table 4). The nationally high proportion of AO varied markedly between regions and was much higher in the eastern regions than in the western regions. Menopause was associated with being more prone to AO (vs. concordance), although much less so after adjustment. Also, being in the third tertile of parity (vs. the first) slightly increased the likelihood of AO. There were no marked associations with socioeconomic factors, except for professional activity, as employee/worker women were somewhat less prone to AO (vs. concordance) than the others. Detailed results for association of OO status with cofactors are not presented here due the small overall prevalence of “overall-only” obesity (1.6%).

**Table 4:** **Abdominal-only obesity by place of residence, physiological, and socio-economic factors among 35- to 70-year-old Tunisian women (complete-case analysis n = 2,633), multinomial logit regression**

		AO: abdominal-only obesity (WC ≥ 88 cm & BMI < 30 kg/m^2^)^a^				
		Unadjusted			Adjusted^b^	
	n	%^c^	RPR^d^	C.I.^e^	RPR^d^	C.I.^e^
**Place of residence**						
**Area**			P = 0.33		P = 0.21	
Rural	1184	27.0%	1	-	1	-
Urban	1449	24.6%	0.9	0.7-1.1	0.8	0.6-1.1
**Region**		P = 0.0004			P < 0.0001	
South-West	366	16.6%	1	-	1	-
Center-West	433	19.2%	1.1	0.7-1.8	1.0	0.6-1.7
North-West	440	20.5%	1.3	0.8-1.9	1.2	0.8-2.0
South-East	384	34.1%	2.5	1.6-3.9	2.6	1.6-4.1
Center-East	366	25.0%	1.6	1.0-2.5	1.7	1.1-2.7
North-East	326	30.0%	2.0	1.3-3.2	2.3	1.5-3.6
Greater Tunis	318	28.1%	1.8	1.2-2.8	2.5	1.5-4.0
**Physiological factors**						
**Age**		P = 0.0017			P = 0.88	
35-44 y.	886	21.7%	1	-	1	-
45-54 y.	937	25.3%	1.2	0.9-1.6	1.0	0.7-1.4
55-70 y.	810	31.0%	1.6	1.2-2.1	1.1	0.7-1.6
**Menopause**		P = 0.0004			P = 0.093	
No	1242	21.8%	1	-	1	-
Yes	1391	29.4%	1.5	1.2-1.8	1.3	0.8-1.5
**Parity**		P = 0.0023			P = 0.061	
1^st^ tertile (0-3)	772	22.0%	1	-	1	-
2^nd^ tertile (4-5)	819	24.1%	1.1	0.8-1.5	1.1	0.8-1.5
3^rd^ tertile (6+)	1042	30.7%	1.6	1.2-2.0	1.4	1.0-2.0
**Socio-economic position**						
**Marital status**		P = 0.46			P = 0.79	
Other	443	27.3%	1	-	1	-
Married	2190	25.0%	0.9	0.7-1.2	0.9	0.5-1.6
**Education**		P = 0.13			P = 0.97	
No formal schooling	1550	27.7%	1	-	1	-
Primary school	769	24.0%	0.8	0.6-1.1	1.0	0.7-1.3
Secondary or more	314	21.3%	0.7	0.5-1.0	0.9	0.5-1.6
**Professional activity**		P = 0.0095			P = 0.038	
Not working/Retired	2142	27.1%	1	-	1	-
Employee/worker	376	18.4%	0.6	0.4-0.9	0.6	0.4-0.9
Upper/Intermediate	115	22.0%	0.7	0.4-1.3	1.0	0.5-1.7
**Household welfare index** ^**f**^		P = 0.67			P = 0.94	
1^st^ quintile	710	25.5%	1	-	1	-
2^nd^ quintile	657	27.6%	1.1	0.8-1.5	1.1	0.8-1.5
3^rd^ quintile	568	25.9%	1.0	0.8-1.4	1.0	0.7-1.4
4^th^ quintile	385	25.1%	1.0	0.7-1.4	0.9	0.6-1.4
5^th^ quintile	313	22.5%	0.9	0.6-1.2	0.9	0.5-1.5

^a^Vs. being a concordant subject (i.e., both abdominal and overall obese or neither abdominal nor overall obese). Results for the second response variable category (OO, overall-only obesity: WC <88 cm & BMI ≥ 30 kg/m^2^) are not presented owing to the small overall proportion of OO subjects (1.6%).

^b^Association of response variable with each place of residence, physiological, or socio-economic variable adjusted for all other variables in column 1.

^c^Prevalence proportion (weighted estimates).

^d^RPR: for category of cofactor vs. reference category (for which RPR = 1), crude or adjusted Relative Prevalence Ratio of being AO, i.e., having abdominal-only obesity vs. being a concordant subject (i.e., both abdominal and overall obese or neither abdominal nor overall obese).

^e^0.95 confidence interval taking into account sampling design.

^f^Increasing household welfare level from 1^st^ to 5^th^ quintile.

## Discussion

### Much higher prevalence of abdominal than overall obesity

Based on a large national random sample of Tunisian women, we found a much higher prevalence of abdominal than overall obesity, similar to results in the few other large-scale studies using national WC data in the MENA region, e.g., Iran [19] or Oman [20] (although not in comparable age classes). Originally, the 88 cm WC “high-risk waist circumference” cut-off value was chosen by the World Health Organization to correspond to a BMI of 30, on the basis of a study in the Netherlands [21]. The large discrepancy in the prevalence of abdominal vs. overall obesity in our study could then result from increases in WC across the whole BMI range over the last decades, as reported in other settings [6]. There could also be ethnicity issues [9], and some authors have proposed a different cut-point of WC ≥ 85 cm to define abdominal obesity among Tunisian women [22]; but, if applied, this would result in an even higher prevalence of abdominal obesity (n = 2964, 68.4% [65.7-71.4]). Other anthropometric indices have been put forward to assess abdominal adiposity, e.g., the waist-to-hip ratio (WHR) ≥0.85, which would result in a similarly higher prevalence of abdominal (n = 2961, 56.2% [53.4-59.0]) vs. overall obesity, or waist-to-height ratio (WHtR) ≥0.6, which was proposed more recently [23], with which the prevalence of abdominal obesity would be lower (n = 2964, 42.6% [39.9-45.4]). Some authors have reported larger seasonal variations in WC vs. BMI, with the difference between the proportion of abdominal vs. overall obesity being somewhat higher in winter than in summer, although in a very different context [24]. However, the present survey took place in the summer months and the discrepancy we observed between the proportion of abdominal and overall obesity was 10-fold that attributed to seasonal variation by these authors.

### One women in four has abdominal-only obesity

The higher prevalence of abdominal vs. overall obesity was mostly due to the high proportion of AO women. Physiological, socio-economic, or lifestyle factors affecting overall obesity in LMIC are well documented [1,25], and some studies deal with correlates of WC, but evidence for why one would preferentially develop abdominal but not overall obesity is not plentiful. As we studied women aged 35 to 70, age could be a factor, since it is thought to be related to preferential accumulation of abdominal fat [26], but even among the younger women in our study, one out of five had AO. The relatively high proportion of postmenopausal women in this population could also be involved, as menopause has been shown to be linked to an accelerated accumulation of central body fat [27], but although we also observed a somewhat higher proportion of AO among postmenopausal women, the prevalence was high in both categories. Reproductive history may also be a factor [28], as mean parity in our population was higher than in the population from which the original WC cut-points were derived [21]. But increases in WC (adjusting for BMI) have been observed in other countries independently of higher parity [29], and also we observed a quite high proportion of AO women even in the lowest parity category. A few authors have suggested that lifestyle factors such as sedentary behavior, high energy intake, total and type of fat intake, or lack of sleep could be linked to abdominal obesity independently of overall obesity, but evidence is generally scarce [30-32]. This could nevertheless be in accordance with the nutrition transition that Tunisia is experiencing [13,33]. Smoking [34,35] or alcohol consumption [31] have also been suggested, but these factors concern a tiny minority of Tunisian women. History of nutrition over the life course such as rapid infant weight gain [36] or exposure to severe undernutrition during the prenatal period [37] have been hypothesized to shift body fat distribution toward abdominal adiposity. They could be significant factors behind the large proportion of AO women in our study, especially in this population with birth dates ranging from 1935 to 1970 (a time frame which includes the troubled period before World War II, the war itself, and the pre- and postindependence periods). Finally, genetics or epigenetics are of course also possible factors [38], with genetic variability of adipose tissue deposits, including anatomical location, possibly interacting with past exposure to various burdens of infectious diseases [39].

### Socio-economic pattern of abdominal-only obesity is weak

On the whole, we found a marked but mostly similar socio-economic pattern of overall and abdominal obesity, and as for the concordance of the two types of obesity at the subject level, almost no independent association of socio-economic factors with AO status, with the exception of a rather mild association with profession. As discussed by some authors [13,40], women working outside the home may benefit from factors related to social and intra-household roles, such as reduced food stimuli, exposition to a healthier lifestyle, or desiring nicer body image, all of which would generally render them less prone to obesity (especially those with a higher job level). Nevertheless, the association of professional activity with abdominal-only obese status would appear to be a somewhat different issue; indeed, as we observed in the present study, only those with a “worker/employee” type of job appeared to be less prone to abdominal-only obesity than nonworking women, while it was not so for the “upper/intermediate” category. This could be linked to the possible association of WC with physical activity (independently of BMI) [30]. Indeed, the employee/worker category that initially grouped “lower-level” jobs with regard to socio-economic position, *de facto* comprises mostly “manual” jobs (detailed data not shown).

### Marked geographic variability of abdominal-only obesity

The unadjusted association with urban/rural area of residence was quite strong as often observed among women in LMIC [5], but was observed similarly for both types of obesity. Thus there was no association of urban vs. rural of residence with AO status, either adjusted or unadjusted. Concerning geographic variability, women from the more developed eastern regions were both more overall and abdominal obesity-prone than women from the less developed western regions, as was also observed for Tunisian adolescents [41]. However, as observed in some other countries, regional variability was much larger for abdominal obesity [42]. There was a much higher prevalence of AO status in the eastern vs. western regions, and this could result from a contextual effect linked to the general level of obesity (higher in the eastern regions) [43]. However, although the prevalence of both types of obesity was also much higher in urban vs. rural areas or in the higher vs. lower quintiles of welfare, no association between AO and these two factors was found.

Regional differences in lifestyle factors could also be involved. However, there is ample evidence that in such a nutrition transition context, there are huge differences in lifestyle factors, e.g., between the urban and rural environment or between different levels of household welfare (including evidence in Tunisia, although in a different age class [33]), but we did not observe any association of the urban/rural variable or the household welfare proxy with AO. Also, in such a nutrition transition situation, these lifestyle factors are to a great extent determined at a higher level of causation, by area of residence (urban vs. rural) or subject- and/or household-level socio-economic factors that are adjusted for in regional comparisons. One would need to hypothesize that residual adjusted differences in lifestyle among regions could explain the large residual regional contrasts in rates of AO. This is all the more unlikely since, as mentioned above, evidence linking these factors to abdominal obesity independently of BMI is quite scarce. It is also generally acknowledged that these factors contribute substantially less to variation in fat distribution than nonmodifiable factors such as ethnicity and genetics [44].

Indeed, over the course of history, many different populations have mixed and been assimilated to varying degrees in what is currently defined as Tunisian territory. Thus, beyond the diverse cultural influences, the relatively small population of 10 million Tunisians is also genetically quite heterogeneous, including genetic features linked to nutrition transition-related NCDs such as type 2 diabetes [45-47]. Such genetic differences could also partly explain regional differences in concordance between abdominal and overall obesity [38]. This would need to be confirmed by appropriate genetic assessment of the geographic disparities.

Regional differences in the history of nutrition over the life course and exposure to severe undernutrition during the prenatal period [36,37] (discussed above with respect to the high overall proportion of AO) may also explain the regional variability of the rate of AO. They could be significant factors, especially in this population whose birth dates ranged from 1935 to 1970, during which the eastern regions underwent more rapid socio-economic development than the western regions. Intergenerational and/or epigenetic factors could also be involved.

Although there is yet no definitive epidemiological evidence, endocrinal disruptors have been hypothesized to be specifically linked to abdominal obesity due to effects on hormonal factors, which, in turn, may influence lipogenesis toward more abdominal fat accumulation [48,49]. The fact that the more developed and industrialized regions of the east had the highest proportions of AO women would be in accordance with such a hypothesis, but data on endocrine disruptors in Tunisia are almost nonexistent.

### Strengths and limitations of the study

The cross-sectional design has limitations regarding the analysis of the dynamics of body fat distribution and its cofactors over the life course [50]. Like in a number of studies pertaining to risk factors of chronic diseases, the 35- to 70-year age class was chosen, but not having included younger adults is a limitation. Like in most large-scale epidemiological studies, overall and abdominal adiposity were assessed by anthropometric proxies only. Apart from issues related to measurement techniques [51], one drawback of WC is that it does not distinguish between different types of abdominal fat accumulation, such as visceral vs. subcutaneous adipose tissues that may be linked to adverse health outcomes in different ways [52,53]. Beyond our characterization of body shape as AO or OO from the internationally acknowledged BMI and WC, some authors have proposed specific indices, such as the ABSI (a body shape index), whose use is not yet standard [54]. Lifestyle factors such as dietary intake or physical activity were not adjusted for in the models, but as discussed above, in such a nutrition transition context these factors are mostly determined at a higher level of causation by individual or socio-economic factors, and these were adjusted for in our comparisons. To our knowledge, this is the first large-scale study in a nutrition transition situation that compares abdominal and overall obesity both at the population and subject level based on a national representative sample. Using multinomial regression to analyze AO and OO status vs. concordance at the subject level and their relationships with environmental, physiological, and socio-economic factors is also original.

## Conclusion

In a typical nutrition transition situation in the MENA region based on a large national sample, we found a much higher prevalence of abdominal than overall obesity, with one in four women having abdominal but not overall obesity. We observed few associated individual or socio-economic factors except for a marked geographic variability of abdominal-only obesity, possibly linked to genetic, epigenetic, or developmental origin differentials between regions. This discrepancy must be taken into account for the assessment of health risks related to obesity and NCDs at the national level and in the management of regional health inequalities in this population. Further, this study underlines the need to include assessments of both abdominal and overall obesity in large-scale epidemiological assessments of the burden of obesity and its correlates in LMIC, especially among the women of this MENA region. The causes of the highly prevalent abdominal-only obesity status require further investigation, as abdominal fat accumulation seems predictive of adverse health outcomes independently of overall corpulence, and in some countries, its prevalence seems to be increasing independently of BMI [29].
